# Emerging Significance and Therapeutic Potential of Extracellular vesicles

**DOI:** 10.7150/ijbs.59296

**Published:** 2021-06-16

**Authors:** Ruhua Luo, Mengmeng Liu, Tiantian Tan, Qian Yang, Yue Wang, Lianhui Men, Liping Zhao, Honghua Zhang, Shuling Wang, Tian Xie, Qingchang Tian

**Affiliations:** 1 College of Pharmacy, School of Medicine, Hangzhou Normal University, Hangzhou, Zhejiang 311121, China.; 2 Key Laboratory of Elemene Class Anti-Cancer Chinese Medicines; Engineering Laboratory of Development and Application of Traditional Chinese Medicines; Collaborative Innovation Center of Traditional Chinese Medicines of Zhejiang Province, Hangzhou Normal University, Hangzhou, Zhejiang 311121, China.

**Keywords:** Extracellular vesicles, Exosomes, Tumor-derived exosomes, Delivery vehicles

## Abstract

Extracellular vesicles (EVs), are membrane-bound vesicles that have many advantages over traditional nanocarriers for drug and gene delivery. Evidence from recent studies indicate that EVs have therapeutic capability with chemical or biological modification. Tumor-derived exosomes (TEXs) were used as a new type of antigens or tumor vaccines in anti-tumor immunotherapy. With superior characteristics, modified EVs were applied to loaded and delivered synthetic drugs, silencing RNA, and microRNA for treatment. Different surface functionalization strategies have been proposed to improve the therapeutic functions of EVs. Appropriately modified EVs for disease intervention provide new avenues for effective clinical treatment strategies. Therefore, this review aimed at elucidating the therapeutic functions of EVs to generate new ideas for treatment and to unlock their hidden potential in translational medicine.

## Introduction

Cancer is a global human health problem that is associated with severe pain and heavy economic burdens to patients and their families 1. Studies on EVs have elucidated on new strategies for cancer diagnosis and treatment. In recent years, nanomaterials for drug delivery have played an important role in the treatment of cancer 2. However, they are associated with certain limitations. Therefore, studies are evaluating the value of EVs in the development of a comprehensive drug delivery system and as a circulating biomarker in cancer diagnosis 3. The International Society for Extracellular Vesicles (ISEV) recognized the potential of EVs as treatments for cancer. EVs or exosome nanovesicles (NVs), which combine the characteristics of cells and nanocarriers, are clinically used in tumor diagnosis 4, and targeted anticancer therapies, such as breast 5, pancreatic 6, lung 7, and liver cancers 8.

In guidelines released by the ISEV, depending on their biogenesis and generation pathways, EVs can be broadly classified into three subpopulations: exosomes (EXOs, 30 to 200 nm in size), microvesicles (MVs, 200-1000 nm), and apoptotic bodies (ABs, larger than 1000 nm) 9. EVs are extracellular nanovesicles secreted by various kinds of cells including dendritic cells (DC) 10, mesenchymal stem cells (MSCs) 11, neural cells 12, epithelial cells 13, and a variety of tumor cells 14. Interestingly, EVs are also distributed in serum, urine, saliva, or any other body fluids 15, 16. They contain and tranfer diversified bioactive molecules include lipids, nucleic acids, and various proteins inside or on the surface of EVs, such as receptors, enzymes, transcription factors, and extracellular matrix proteins into adjacent or distant cells through the systemic circulation, participating in intracellular and intercellular communication, and regulate host tumor cell interactions 17. As a general rule, of the targeting and fusion proteins present on the surface of EVs, the most abundant are integrins and Tetraspanins. The members of the tetraspanin family, such as CD9, CD63, CD81 and CD82, regulate intercellular signal transduction 18, 19. As EVs contain biomolecules active from maternal cells, they can regulate function, fate, and shape of target cells, participating in different pathological and physiological conditions 20, 21. Conserved proteins participate in cytoskeleton formation (β-actin, myosin and tubulins), metabolism (glyceraldehyde 3-phosphate dehydrogenase) and protein folding (Hsp70) 22, 23. Cell-type-specific proteins such as notch ligands, β-Catenin, Wnt as well as intercellular cell signaling mediators mainly involved in cell signaling pathways such as TNF-α, TGF-β, and IL-1β 24. Of course, EVs also contain nucleic acids, including mRNA, miRNA, DNA, and mitochondrial DNA (mtDNA). These nucleic acids are mainly involved in inflammation and as diagnostic biomarkers in tumors 25, 26. In recent years, scientists have paid more attention to EVs due to their key roles in biological systems.

Exosomal liposome structures allow the loading of various drugs, which enhances drug delivery to specific targets. EVs, as carriers, can transport their cargos to particularly intracellular locations in a target-specific manner across the plasma membrane. As natural nucleic acid and protein carriers, they have been used as vectors for targeted delivery of these molecules 27, 28. Based on their endogenous performances and multifunctional properties, EVs have a clinical potential for the development of efficient therapeutic options for cancer.

Evidence from recent studies indicate that EVs have therapeutic potential in tumors 29, neurological diseases 30 and immune diseases 31. For instance, exosomes are not only used as carriers for tumor treatment drugs 32, but also as tumor immunotherapy, especially for those extracted from tumor cells robustly eliciting anti-tumor immune responses 33, 34. Given their low immunogenicity and high biocompatibility, exosomes can stably stay in the circulatory system for longer periods. Targeted administration of exosomes carrying drugs to tumor lesions can double the anti-tumor effect of such drugs. When used as carriers, EVs not only provide intrinsic immunomodulatory activity, but also have many advantages such extended circulation half-life, high biocompatibility, transfection efficiency, low immunogenicity and minimal reversion to virulence, over traditional nanocarriers for drug and gene delivery 34. Hence, EVs may provide opportunities to enhance or broaden the innate therapeutic capability with chemical or biological modification.

## EVs in cancer immunotherapy

Immunotherapy has shown promising prospects in the treatment of cancers, and EVs are currently applied in tumor immunotherapy. EVs have several advantages and appear to be a highlight of new pattern for cancer immunotherapy at present 35. The use of EVs as a new type of antigens in anti-tumor immunotherapy has raise some concerns. By reactivating the patient's immune system, EVs exploit autoimmune cells, especially CD8+ T cells, to generate anti-tumor responses 36. Although the potential of these therapies is widely known, there is still significant room for improvement. Associated immune checkpoint therapies tend to be ineffective and severe autoimmunity could also occur. Many tumors, due to genetic, biological and other factors make it unlikely that some patients will respond to these therapies. To overcome some of these obstacles, innovative approaches with lower toxicity and providing more frequent and durable response are needed. One such treatment is the use of nanoparticles, particularly EVs. Research has shown that TEXs potentiate PD-L1 function and suppress immune response 37. In addition, in patients with melanoma receiving PD-1 blockade, PD-L1 levels in exosomes are correlated with tumor burden and treatment response. Although it is unclear whether the exosomal PD-L1 is directly related the failure of anti-PD-1 therapies, PD-L1-containing exosomes may be regulators and biomarkers of resistance to treatment.

Surface engineered EVs can actively participate in tumor immunotherapy. CD47, a "don't eat me" signal, limits the ability of macrophages to engulf tumor cells by binding to SIRPα 38. Therefore, the exosomes carrying SIRPα variants may serve as immune checkpoint blockers, thereby antagonize the interaction between CD47 and SIRP. This will enhance tumor phagocytosis and exert effective anti-tumor T cell response 39. Although the immunotherapeutic effects of dendritic cell vaccine have been reported, it is still unclear how tumor-associated exosome-based dendritic cell (DC) vaccine-based anti-tumor immunity can be induced to achieve antitumor effects 40. Studies have shown that dendritic cell-derived exosomes (DEX) can regulate the immune responses to cancer 41. DEXs stabilizes vesicles, and are not easily degraded or inactivated. Research on DEXs has shown that DEX is rich in membranous proteins such as major histocompatibility complexes class I (MHC class I), MHC class II molecules, CD63, CD81, and integrin, and has a strong immune activation effect 42. The use of EVs as tumor vaccines has shown promising anticancer effects *in vivo*, and results from clinical trials on EVs have been feasible. A novel DEX vaccine with antigens and matured with either the TLR-3 ligand induced robust activation of melanoma-specific CD8(+) T cells and the recruitment of cytotoxic CD8(+) T cells, NK and NK-T cells to the tumor site, resulted in significantly reduced tumor growth and enhanced survival 43. A phase I clinical trials to test DEXs loaded MAGE tumor antigens in patients with non-small cell lung cancer (NSCLC) showed that some patients experienced long term stability of disease and activation of immune effectors 44. A phase II clinical trial testing the clinical benefit of DEX loaded with MHC class I- and class II-restricted cancer antigens as maintenance immunotherapy confirmed the capacity of DEX to boost the NK cell arm of antitumor immunity in patients with advanced NSCLC 45. DEX maintain the key functions of DCs in their ability to present tumor-associated antigens (TAAs) and to activate TAA-specific immune responses 46. To improve immunogenicity, exosomal antigen-adjuvant co-delivery systems have been engineered for cancer immunotherapy. Exosomes derived from genetically engineered tumor cells containing endogenous tumor antigens and CpG DNA with immunostimulatory have been found to confer anti-tumor effects 47. Therefore, blocking the biogenesis and release of TEX seems to be a potential anti-tumor strategy. GW4869, an inhibitor of nSMase2, has been found to block ceramide synthesis. Treatment with GW4869 reduced lung metastasis in tumor-bearing mice. A combination of GW4869 with cisplatin and gefitinib provided strong antitumor effect 48.

## EVs as drug carriers in cancer

Exploitation of EVs in anti-tumor research has gained momentum in recent years, and many studies have evaluated EVs -related antitumor effects. Compared with EVs, delivery of drug formulations such as liposomes 49, micelles 50, and microcapsules 51 using traditional carriers is limited in clinical application, due to immune rejection, low drug loading, and poor targeting. EVs, as carriers, do not elicit immune responses in the bloodstream like other nanoparticle formulations 52. Given their superior characteristics to natural or synthetic polymers and liposomes, many studies (Table 1) have reported that synthetic drugs, silencing RNA, and microRNA can be loaded into modified EVs and delivered into tumors yielding good results.

The most common treatments for malignant tumors are chemotherapy, radiation surgery, or combination therapy. Cancer targeted therapy is an emerging treatment approach in anti-tumor therapy. However, the success of targeted therapy requires a drug delivery carrier with low immunogenicity and low toxicity. Doxorubicin (DOX) was loaded into exosomes through electroporation, and delivered to breast cancer tumor cells by engineered exosomes leading to effective targeting 53. The main advantage of using exosomes to carry biological molecules over other nanoparticles is that they possess natural ability to carry bio-related molecules such as nucleic acid drugs and can activate the immune system. Ohno *et al.* modified exosomes with a GE11 peptide that specifically binds to epidermal growth factor receptor (EGFR) and was loaded with let-7a miRNA, a regulator that reduced cell division and altered the cell cycle 54. A synthetic multivalent antibodies retargeted exosome (SMART-Exosome) was designed to cross-link tumor cells with T cells and induce a strong immune response which effectively killed tumor cells *in vivo* and *in vitro* 55. Although numerous drugs have been designed for prevention of cancer progression and suppress tumor development, of the efficacy of such drugs is limited by the low bioavailability and high toxicity. A highly biocompatible tumor cell-targeted delivery system has been designed to deliver imperialine (a less toxic anti-cancer agent) into NSCLC cells using exosome-like vesicles (ELVs) 56. Given the high expression of alpha 3 beta 1 on NSCLC cells, they modified integrin alpha 3 beta 1-binding octapeptide cNGQGEQc to create an ELV platform for targeting tumor. This platform not only significantly improved accumulation and retention of imperialine in the tumor, but also exhibited extremely low systemic toxicity *in vitro* and* in vivo*. Natural compounds such as anthocyanins (Anthos) found in berries are limited by low permeability and oral bioavailability. Munagala *et al.* delivered exosomes loaded with anthocyanins from raw milk to mice with lung cancer 52. They reported that exosomes provided an effective alternative for oral delivery of Anthos for efficient systemic delivery and robust bioavailability. For nervous system tumors, the blood-brain barrier (BBB) limits the entry of therapeutic drugs into the brain. Yang *et al.* tried to further their zebrafish studies pass through loading siRNA in the exosomes 57.

## EVs for gene therapy

**Figure 1:**
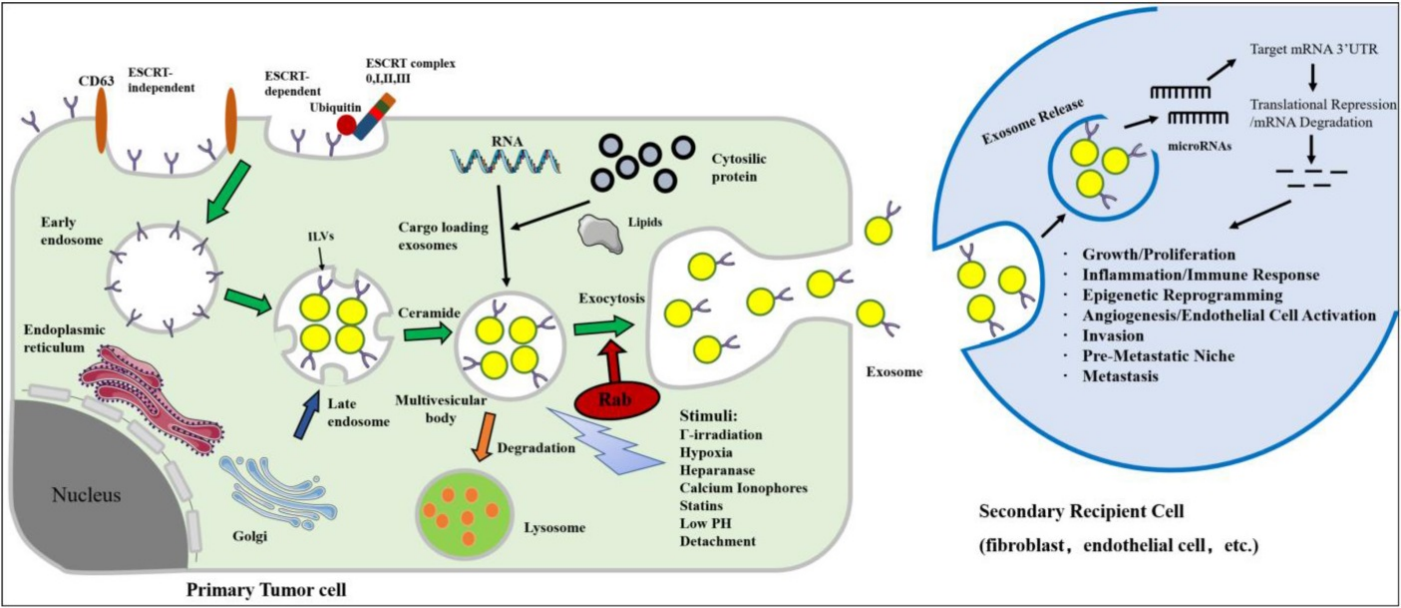
Biogenesis, secretion and uptake of tumor-derived exosomes in the tumor microenvironment. Inward invagination of the cell wall mediated by either ESCRT complex with the help of ubiquitin (ubiquitinated ESCRT-dependent way) or ceramide-triggered inward budding (ESCRT-independent way) in the presence of CD63. Exosomes are formed by the inward budding of the multivesicular body (MVB) membrane in the form of intraluminal vesicles (ILVs). Eventually, exosomes are secreted in exocytic MVBs following fusion of MVBs with the cell membrane, a process that depends on Rab GTPases. MVB may undergo degradation by lysosome for recycling its content. The secretion of exosomes can be stimulated by various chemical, environmental, and mechanical stimuli, such as Γ-irradiation, hypoxia, low pH, etc. Exosomes can release their microRNA cargo. The transferred microRNAs are functionally active and can regulate gene expression in recipient cells by post translationally modulating the expression of target mRNAs, leading to mRNA degradation or instability. MicroRNA dependent gene regulation can activate various processes involved in tumor development and progression.

Donor cells have also been engineered to isolate qualified exosomes that contain the gene or drug of interest 58. Exosomes are secreted from engineered cells through the endosomal pathway, and this pathway have been hijacked with viruses and used for superior delivery of RNA *in vivo* 59. Many studies have exploited the genetic material naturally carried by foreign bodies, and many cancer-based studies have investigated exosomes using microRNAs. Thus far, exosome-derived microRNAs, through target gene transcriptional repression, have the demonstrated ability to induce cell migration, inflammation, immune responses, angiogenesis, invasion, pre-metastatic niche formation and metastasis (Figure 1). Masaki *et al.* found that miR-199a-3p-Exo suppressed c-Met expression, a direct target of miR-199a-3p, leading to the inhibition of cell proliferation and invasion 60. The engineered exosomes were utilized to simultaneously deliver the anticancer drug 5-Fu and the Mir-21 inhibitor oligonucleotide (Mir-21I) to cancer cells expressing human epidermal growth factor receptor-2 (HER2) 61. This effectively reversed drug resistance in 5-FU-resistant colon cancer cells and significantly enhanced cytotoxicity. These studies highlight the potential of exosomes encapsulated with tumor suppressor miRNAs in the treatment of cancer.

Some *in vivo* studies have also been conducted with satisfactory results. Introduction of T7-exo and antisense miRNA oligonucleotides against miR-21 (AMO-21) via tail vein effectively introduced AMO-21 into the brain and reduced Mir-21 levels in glioblastoma in rats 62. Downregulation of miR-21 by AMO-21 induced the expression of PDCD4 and PTEN in tumors, thereby decreased the tumor size. In a study, miR-129-3p directly inhibited the expression of SUMO-activated enzyme subunit 1 (SAE1) by targeting 3'UTR and also suppressed the zoylation of XRCC4, leading to more DNA damage in gastric cancer cells and inhibition of the proliferation, migration and invasion of gastric cancer cells 63. The effect of exosomal encapsulated miRNAs on tumor chemical sensitivity was also investigated. Intra tumoral injection of miR-122-containing exosomes combined with sorafenib significantly reduced the weight and volume of tumors, indicating that exosomes from miR-122 adipose derived MSCs (AMSC) may enhance the sensitivity of human hepatocellular carcinoma (HCC) cells to chemotherapy 64. The enhanced efficacy of engineered exosomes (iExosomes) in targeting oncogenic KRAS compared to liposomes has also been studied 65. A key driver of common mutations in pancreatic cancer is the mutational form of GTPase KRAS. Application of a targeting method called RNA interference (RNAi) successfully inhibited tumor growth and significantly increased overall survival of mice with pancreatic cancer.

## Modification of EVs for targeted delivery

**Figure 2:**
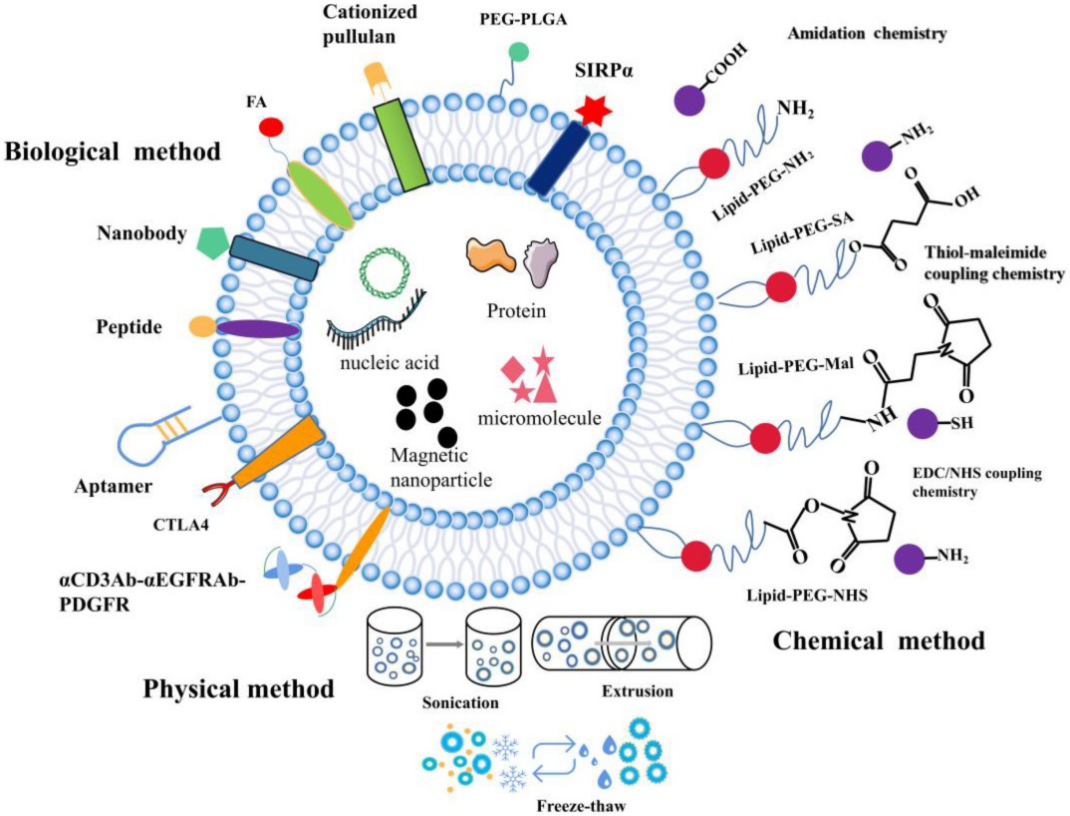
The surface functionalization of EVs. A schematic illustration of the physical, biological, and chemical strategies used for surface functionalization of exosomes.

The composition of EVs can be modified at the cellular level. EVs from various biological sources can be modified after isolation when cell-derived exosomes are unable to meet the requirements. While preserving the membrane integrity of exosomes, functional fluorescent tags 102, imaging probes 103, immuneactivators 104, and targeted therapeutic agents can be added 105. Modified EV surface structures allow *in vivo* imaging and tracking of EVs. Although EVs have been shown to be useful *in vitro* anticancer drug carriers, this is not the case *in vivo* given their non-specific toxicity and off-target effects similar to those observed in conventional chemical drugs 106. EVs need to be functionalized with specially designated parts to optimize their transmission properties. To improve the therapeutic functions of EVs in cancer treatment, different surface functionalization strategies have been proposed as shown in Figure 2.

## Surface engineering of EVs

To further increase the functions of EVs, different surface engineering strategies have been explored, which can be roughly divided into three main approaches: physical, chemical, and biological approaches.

### Physical methods

In this approach, physical approaches such as ultrasonic treatment, extrusion, and freeze-thaw are used to temporarily destroy lipid structures. Once the structures are removed, the vesicles spontaneously re-assemble into their natural structure. Sagar *et al.* hybridized macrophage-derived exosomes with liposomes through freeze-thaw method which allowed membrane fusion to form hybrid immune exosomes for doxorubicin treatment of breast cancer 107. Since freezing and thawing can lead to denaturation of proteins, to avoid this, they applied extrusion method to promote membrane fusion. Natural killer cell exosomes (NKsome) were prepared by simple liposome membrane extrusion technology 108. The engineered NKsome successfully retained the targeted proteins associated with the NK cell membrane on its surface and showed higher affinity for cancer cells. Moreover, doxorubicin-loaded NKsome showed good homing efficiency *in vivo*, and its plasma retention time was extended by 18 h providing strong therapeutic effects. A biomimetic nanostructure (BNS) with a multimodal imaging system was designed to coat polymer nanoparticles with natural killer cell membrane (NKM), near-infrared ray (NIR) fluorescent dye and Gadolinium (Gd) conjugated magnetic resonance imaging (MRI) contrast agent 109, which allowed *in vitro* and *in vivo* tracking 110. The physical approach of surface functionalization allows for the simple reagent free functionalization of exosomes. Compared to biological and chemical methods, physical method does not require additional reagents or cell-based systems.

### Chemical methods

Chemical approaches for surface functionalization involve direct use of chemical reagents to anchor functional parts to the surface of exosomes. The transmembrane protein portion of phospholipids or amine/carboxylate on surface of exosomes can be directly functionalized with various functional groups. Alternatively, functional phospholipids obtained from exosomes can be added to exosomes by simple incubation using a hydrophobic insertion strategy. Firstly, lipid functionalization of exosomes sealed with maleimide is achieved via the hydrophobic insertion strategy. A hydrophobic insertion strategy in which maleimide-terminated DSPE-PEG-Mal is used as a labeling probe, which is labeled with fluorescent dye containing maleimide for monitoring of cell communication 111. Secondly, azide can be linked with cargo-conjugated dibenzobicyclooctyne (DBCO) through azide-alkyne cycloaddition. Wang *et al.* combined metabolic markers of newly synthesized proteins or polysaccharides/glycoproteins from secreting exosomal cells with chemically active azido groups, modified and functionalized exosomes through bioorthogonal click conjugation 112. Lastly, copper-catalyzed azide-alkyne cycloaddition is another click chemistry strategy that can be used for surface functionalization of azide-functioning exosomes. Exosomes were chemically crosslinked with alkyne groups by carbodiimide and coupled to a model azide, fluoroazide 545. The coupling had no by-effect on the size of exosomes, nor did it alter the degree of adhesion/internalization of exosomes to recipient cells. This technique is superior to other exosome-labeling methods and is likely to find widespread application in exosome research 113. These chemical methods are widely used to functionalize biomolecules because they are easy, fast and compatible with biomolecules.

### Biological method

Studies show that different surface functionalization strategies can enhance targeting of exosomes by adding targeted peptides or ligands to EV surfaces. Expression of a well-characterized exosomal membrane protein (Lamp2b) on engineered exosome DCs fused to αv integrin-specific iRGD peptide has been reported to promote tumor targeting 53. EVs modified with GE11 peptide, a synthetic peptide that binds specifically to EGFR, could efficiently deliver let-7a miRNA to EGFR-expressing xenograft breast cancer tissues in mice, leading to a marked inhibition of tumor growth 114. Besides, specific targeting molecules such as folic acid (FA), iron oxide, and aptamer have been used to modify EV. Aptamers with high affinity and specificity for their targets are often considered as substitutes for antibodies in targeted delivery. Targeted delivery of miR-21 to leukemia cells was achieved by modifying exosomes with the cholesterol-conjugated aptamer AS1411 115. Most tumors are angiogenic and produce exosomes, and novel strategies targeting exosome induced angiogenesis can reduce tumorigenesis 116. Corrado *et al.* reported that carboxyamidotriazole orotate (CTO) targets tumor exosomes promoted IL-8 expression and cell adhesion of endothelial cells (ECs). Therefore, CTO inhibits the effects of these exosomes on ECs-chronic myelogenous leukaemia (CML) interaction and the migration of ECs, suppresses angiogenesis induced by exosomes 117. This resulted in enhanced specific targeting of cancer cells and effective inhibition of tumor growth. Folate receptor (FR) is a glycosylphosphatidylinositol glycoprotein anchored on the cell surface, which is overexpressed in many epithelial malignancies, including ovarian, breast and lung cancers. Erastin-loaded exosomes labeled with FA were used to target triple negative breast cancer (TNBC) cells overexpressing FA receptors 71. Tissue-specific delivery may also be achieved by loading exosomes with magnetic nanoparticles. It has been reported that exosomes anchored with cell-targeted peptides (CPPs) and TNF containing superparamagnetic iron oxide nanoparticles improve tumor targeting in external magnetic fields and inhibit tumor growth 118. Surface-functionalization approach promotes expression of targeted cargo on exosomes surface. Apart from peptides, incorporating targeting proteins containing nanobodies to exosome surface has also been demonstrated as an attractive targeting strategy. Cheng *et al.* linked a rather sophisticated polypeptide composed of two single chain variable fragment (scFv) antibodies targeting CD3 and EGFR on top of the domain of human platelet-derived growth factor (PDGFR) 55. Here, they integrated with EVs by the EV biogenesis process to obtain surface-functionalized EVs with antibodies. Exosomes prepared from Ovalbumin (OVA)-pulsed, activated dendritic cells were modified with anti-CTLA-4 antibody to block this inhibitory molecule and to enhance the specificity of the exosomes toward T cells. This study provides a unique strategy to endow exosomal membranes with the function of anti-CTLA-4 antibody to synergize the efficacy of cancer vaccines and checkpoint blockade on tumors 119.

## Remaining concerns and future perspectives

The extraction, isolation, and identification of tumor cell-derived EVs can be used to elucidate the mechanisms underlying tumor progression and provide potential therapeutic targets for cancer patients. Secondly, EVs can be loaded in different antitumor drugs to treat cancer. Lastly, with increasing interest in tumor immunotherapy, EV immunomodulatory properties mainly include regulating antigen presentation, immune surveillance, and immune activation. It is likely that a new approach to tumor immunotherapy will be revealed through an in-depth study of the molecular mechanisms underlying the interaction between EVs and immune cells.

As intermediates of intercellular communication, EVs have heterogeneous and pleiotropic physiological and pathological roles. However, many issues still need to be addressed before its clinical application. Due to tissue and cell specificity, not all tissues and cells express these so-called EV markers. Therefore, further investigation into how to identify EVs is a significant goal for future research. Isolation of purification specific EVs is limited by technical limitations, and specific EVs by the availability of suitable biomarkers as well as expensive techniques. It is necessary that we develop standard and highly efficient methods for EV isolation, purification, characterization, and manipulation that allow these vesicles to be successfully applied in the clinic.

## Conclusion

In the field of nanofabrication, EVs have been widely applied in the diagnosis and treatment of tumors, but the clinical application of exosomes still faces arduous challenges. EVs play important roles in the tumor microenvironment, tumor and non-tumor tissue. Considering their good biocompatibility, tumor targeting and low immunogenicity, EVs have been proven to be potential drug carriers. However, the complex characteristics of exosomes are not thoroughly understood, it is important to comprehensively define the many subtypes of exosomes. Although there are many challenges in the costs, technical challenges, and lack of suitable biomarkers for EVs isolation and purification, more classification and loading mechanisms of exosomes can be exploited in further research to develop efficient exosome-based drug delivery systems. Moreover, EVs have shown great promise in tumor targeting, tumor immunotherapy and inhibition of tumor metastasis. In the future, in-depth research is needed to develop effective tools for efficient drug loading, targeted modification, and studying the mechanisms that drive tumor development and response to drugs. Such efforts will also generate new ideas for the diagnosis and treatment of tumors.

## Figures and Tables

**Table 1:** Overview of Cancer Type, Exosomal Cargo, Loading method and Source of EVs Discussed in This Review.

Cancer type	Drug	Source of exosome	Loading method	Outcome	Ref.
Breast cancer	Paclitaxel; Doxorubicin	Macrophage cells	Sonication	Inhibition of tumor growth	66
	Doxorubicin; miR159	THP-1 cells	Incubation	Silenced the TCF-7 gene	67
	Taxol	Human mesenchymal stroma/stem-like cell	Incubation	Inhibition of tumor metastases and target specificity	68
	siRNA	HEK 293 with surface modification by LAMP2b-DARPin G3 chimeric gene	Transduction	Target specificity; TPD52 gene expression is downregulated	69
	Doxorubicin	Breast cancer cell line and mouse ovarian cells	Electroporation	Inhibition of tumor proliferation	70
	Doxorubicin	Mouse immature dendritic cells	Electroporation	Inhibition of tumor proliferation	53
	Erastin	HFL-1 cell	Sonication	Inhibition of tumor proliferation and metastases	71
	Doxorubicin	J774A.1 cell	extrusion	Increased target specificity	72
	Curcumin	Bovine milk	incubation	Inhibition of tumor growth	73
	Paclitaxel	Macrophage cells	Incubation; electroporation; sonication	Inhibition of tumor growth and metastases	74
	Paclitaxel	Raw bovine milk	Incubation	Inhibition of tumor growth	75
	Berry Anthos	Raw bovine milk	Incubation	Inhibition of tumor proliferation	52
	Doxorubicin	J774A.1 cell	Electroporation	Promote apoptosis and silence target genes	72
Lung cancer	Withaferin A	Bovine milk	Incubation	Reduced tumor growth	76
	Curcumin	Bovine milk	Incubation	Reduced tumor growth	77
	Celastrol	Raw bovine milk	Incubation	Increased drug efficacy and inhibition of tumor growth	78
	Paclitaxel	Macrophage cell	Sonication	Increased target specificity and inhibition of tumor growth and metastases	79
	Doxorubicin-gold nanoparticle conjugate	H1299 and YRC9 cell	Incubation	Reduced cellular toxicity and increased efficient delivery	80
Pancreatic	Oncogenic Kras	Human foreskin fibroblast cell	Electroporation	reduced tumor growth and targeting KRAS	65
	Oncogenic Kras	Bone marrow-derived mesenchymal stemcell	Electroporation	Reduced tumor growth and targeting KRAS	81
	Doxorubicin	Macrophages cell	Incubation	Increased antitumor efficacy	82
Prostate	Paclitaxel	LNCaP and PC3 cell	Incubation	Increased drug cytotoxicity	83
	SPIONS	Human mesenchymal cell	Incubation	Inhibition of tumor proliferation	84
Glioblastoma	Curcumin; STAT3 inhibitor	GL26 cell	Incubation	Reduced tumor growth and increased Target specificity	85
	MiR-124a	Mesenchymal stem cell	Incubation	Silence Forkhead box (FOX)A2 and reduced tumor growth	86
	SiRNA; Paclitaxel or doxorubicin	bEND.3 cell	Incubation	Increased drug cytotoxicity and crossed the BBB	87
	Doxorubicin, paclitaxel	Brain cell	Microinjection	Tumor growth inhibition	87
	miR146b	Mesenchymal stem cell	Incubation	Inhibition of tumor proliferation	88
	miR9	Mesenchymal stem cell	Incubation	Increase in chemosensitivity and tumor regression	89
	Paclitaxel	Embryonic stem cell	Incubation	strong ability to cross the BBB and enhanced targeting	90
Ovarian	Cisplatin	Umbilical cord-derived macrophage cell	Sonication	Increase in chemosensitivity and drug cytotoxicity	91
	miR-199a-3p	Omental fibroblasts of OC patients	Electroporation	Inhibit cell proliferation and invasion	60
Oral squamous cell Carcinoma	Cabazitaxel /TRAIL	Mesenchymal stem cell	Ultracentrifugation and dialysis	Tumor growth inhibition	92
Hepatocellular	miR-31,miR-451a	Plasma	Electroporation	Silence target genes and promote apoptosis	93
	rAAV/AFP	Human peripheral blood dendritic cell	Transfection	Increased drug cytotoxicity	94
	miR-26a	293T cell	Electroporation	Inhibit cell proliferation^95^	95
Melanoma	Ovalbumin	Dendritic cell	Incubation	Tumor growth inhibition	96
Gastric	rMETase	Immature dendritic cell	Electroporation	Tumor growth inhibition	97
Colorectal	5-FU, miR-21	Culture supernatants of THLG-293T or LG-293T cell	Ultracentrifugation	Enhanced the cytotoxicity and reverse drug resistance	98
	miR-128-3p	FHC cell	Ultracentrifugation	Tumor growth inhibition and increase in chemosensitivity	99
	Doxorubicin	Human umbilical vein endothelial cell	Incubation	Tumor growth inhibition	100
Leukemia	miR-21	Plasma	Transfection	Tumor growth inhibition	101

## Abbreviations

EV: extracellular vesicle
TEXs: tumor-derived exosomes
ISEV: International Society for Extracellular Vesicles
NVs: nanovesicles
EXOs: exosomes
MVs: microvesicles
ABs: apoptotic bodies
DC: dendritic cell
MSCs: mesenchymal stem cells
mtDNA: mitochondrial DNA
DEX: dendritic cell-derived exosome
MHC class I: major histocompatibility complexes class I
NSCLC: non-small cell lung cancer
TAAs: tumor-associated antigens
DOX: Doxorubicin
EGFR: epidermal growth factor receptor
SMART-Exosome: synthetic multivalent antibodies retargeted exosomes
ELV: exosome-like vesicle
Anthos: anthocyanins
BBB: blood-brain barrier
Mir-21I: Mir-21 inhibitor oligonucleotide
HER2: human epidermal growth factor receptor-2
SAE1: SUMO-activated enzyme subunit 1
AMSC: adipose derived MSC
HCC: human hepatocellular carcinoma
iExosomes: engineered exosomes
RNAi: RNA interference
MVB: multivesicular body
ILVs: intraluminal vesicles
NKsome: Natural killer cell exosome
NKM: natural killer cell membrane
NIR: near-infrared ray
MRI: magnetic resonance imaging
DBCO: dibenzobicyclooctyne
FA: folic acid
CTO: Carboxyamidotriazole orotate
ECs: endothelial cells
FR: folate receptor
TNBC: triple negative breast cancer
CPPs: cell-targeted peptides
scFv: single chain variable fragment
PDGFR: human platelet-derived growth factor
OVA: Ovalbumin
ECs-CML: endothelial cells-chronic myelogenous leukaemia
